# Impact of Dispensing Services in an Independent Community Pharmacy

**DOI:** 10.3390/pharmacy7020044

**Published:** 2019-05-10

**Authors:** Kaleb Payne, Elizabeth J. Unni, Benjamin Jolley

**Affiliations:** 1 Pharmacy Class of 2019, College of Pharmacy, Roseman University of Health Sciences College of Pharmacy, 10920 South River Front Parkway, South Jordan, UT 84095, USA; kpayne@student.roseman.edu; 2 Department of Pharmaceutical Sciences, Roseman University of Health Sciences College of Pharmacy, 10920 South River Front Parkway, South Jordan, UT 84095, USA; 3 Jolley’s Compounding Pharmacy, 1702 South 1100 East, Salt Lake City, UT 84105, USA; Benjamin@jolleydrug.com

**Keywords:** community pharmacy, dispensing, impact, safety, adherence, cost saving, independent pharmacy, dispensing activities, prescription clarification

## Abstract

With approximately 300 prescriptions dispensed per day in a typical community pharmacy, several activities take place to ensure the safe dispensing of medicines. While some of these activities are common for all prescriptions, others need further activities such as prescription clarification. These activities are important to avoid any potential harm to the patient and improve medication adherence. The objective of this study was to measure the impact of these additional dispensing services in a community pharmacy by evaluating the documented patient notes. Two-hundred patients with annotations on their profiles between 1 July and 31 August 2018 were randomly selected and 322 notes were analyzed. The average number of notes per patient was 1.6. The majority of the notes were about contacting the patient/caregiver for prescription clarification (86.8%). When analyzed based on Medication Therapy Problems, 54.7% were related to adherence and 35.4% to safety. Using the cost saving estimate from the literature for each averted adverse event, these activities resulted in a minimum cost saving of $10,458. This study was able to show the positive impacts that everyday dispensing services in an independent community pharmacy have on ensuring the safe use of medication, thus potentially preventing adverse drug events and reducing health care costs.

## 1. Introduction

More than 7 million patients in the United States are affected by a preventable medication error annually in all health care settings [1]. According to the US Department of Health and Human Services, 24–33% of adverse drug events following discharge from the hospital were preventable [1]. Relatively few studies have been done to determine the rate of medication errors in the community pharmacy setting. Flynn and colleagues, at the US national level, reported dispensing errors at a rate of four errors per day in a pharmacy filling approximately 250 prescriptions a day, with 6.5% of them being clinically significant errors [2]. When converted to national statistics, this accounts for 51.5 million errors each year across the United States [3]. In the UK, the dispensing error rate was determined to be 0.1%, which equals to approximately 2700 errors each day [4]. Another study based on community pharmacies in England and Wales reported the error rate as 22 near misses and four dispensing errors for every 10,000 items dispensed [5]. 

A 2017 study reported that 90% of Americans live within 2 miles of a community pharmacy [6]. With approximately 67,753 community pharmacies, 4.1 billion prescriptions were dispensed in the United States in 2017 [7,8]. This magnitude of prescriptions being processed provides great opportunity for a pharmacy team to carry out dispensing services. The goal of each pharmacist, pharmacy technician, and intern is to ensure that the patient receives optimal medication therapy. This is implemented by carrying out various activities on each prescription. The essential activities that take place while dispensing these prescriptions include entering the prescription into the patient’s profile, making sure that the prescription written is accurate, ensuring that the medication is safe and appropriate considering available information about the patient’s diseases and other medications, filling the prescription, verifying that the final product is correct, and finally dispensing the medication to the patient with counseling. 

However, to prevent medication errors and to improve medication adherence, some prescriptions require further actions such as contacting a physician’s office, insurance company, another pharmacy, or the patient themselves. In addition to ensuring the safe and accurate dispensing of medicines, these additional activities also confirm that the pharmacy is “connecting all the dots” when the patient has multiple providers and medications. For example, contact with a physician’s office will allow for clarification and the correction of doses and administration directions. Communication with an insurance company ensures that the patient can get their medication at the most affordable price possible. Additionally, insurance companies may be contacted for a prior authorization or vacation override. Contacting another pharmacy for inventory reviews and transfers ensures the patient gets their medication promptly. Contact with a patient may be needed to gather additional information about the patient’s health and medication use, or to educate them about the safest way to take their mediation. All these activities require time and effort from pharmacy personnel and are important for avoiding potential harm to the patient and improving medication adherence. 

Many patients are unaware of all the activities that occur to ensure the safety and accuracy of their prescription before it is dispensed. At times, other health care providers such as physicians or nurses are also unaware of all the activities that are carried out on a prescription before it is dispensed. The quantification of these activities carried out on each prescription as well as the impact of these additional dispensing activities are not well documented by the pharmacy profession. At a time when the profession is pushing for provider status and identifying ways to be paid for these core services, it is essential to quantify said services and identify their impact in health care. 

This paper aims to show the impact of the additional dispensing activities carried out by pharmacy personnel including ensuring safe dispensing, avoiding potential adverse drug events, improving medication adherence, and cost savings. The specific objectives of this study were to quantify the number of additional activities carried out in a typical day, categorize the purpose of these activities, and calculate the potential cost savings from these activities. 

## 2. Materials and Methods

The study objective was achieved by evaluating patient notes documented in an independent community pharmacy system to determine what additional activities were performed on each prescription while dispensing. Automated system default notes or reminders on which follow-up action may not have happened were not included. For this study, the additional activities were defined as contacting a physician, insurance company, another pharmacy, or the patient/caregiver. Two-hundred patients with annotations on their profiles between 1 July 2018 and 31 August 2018 were randomly selected. This study was approved as exempt by Roseman University Institutional Review Board. 

The notes were categorized according to reasons that a physician, insurance company, patient/caregiver, or another pharmacy were contacted via email, fax, in-person, or over the phone. Activities involving communication with a physician were assigned as follows: incomplete script, inappropriate dosage, inappropriate instructions, refills, change medicine due to lack of insurance coverage, change brand to generic, medicine too expensive, formulary coverage, change medicine due to drug–drug interactions, change medicine due to allergies/side effects from the prescribed medicine, change medicine due to unnecessary therapy, additional therapy needed, duplicate therapy, medicine recall/backorder, new medication, med-list, and medication error. Tasks involving contact with an insurance company were categorized according to the following reasons: medication change, prior authorization, early refill, eligibility check, vacation supply, workers compensation, and formulary review/process claim. Communication with a patient/caregiver involved activities that were assigned to the following categories: medication change, additional education on the medication, med-synchronization/adherence, reminder to pick up medication (in addition to the regular texts), refill due, prescription clarification, inform of dosage/other change in therapy, safety, call to caregiver, vaccine recommendations, med-list, in-pharmacy counseling, medication error, and prescription profile review. Activities associated with contacting another pharmacy were categorized under prescription transfer. 

Next, using the Medication Therapy Problem (MTP) categories created by the Pharmacy Quality Alliance, each reason that contact was made was appropriately placed under indication, effectiveness, safety, or adherence. The date and time that each note was published, the employee type who performed the activity (such as pharmacist, technician, or intern), the duration spent on each activity (when annotated in patient note), and the care comments published on the patient’s record were all gathered from the pharmacy system and organized on an Excel sheet. A literature review was conducted to determine cost associated with preventing adverse drug events. The total money saved was calculated to demonstrate the impact of dispensing services.

## 3. Results

Between 1 July and 31 August 2018, 2401 patients filled 7732 prescriptions at the independent pharmacy evaluated. At approximately 24 working days a month, this equates to 161 prescriptions per day. In this time period, the pharmacy personnel documented 937 eligible notes on 588 patients. From these 588 patients, an Excel random number generator was used to randomly select 200 patients. These 200 patients had 322 eligible notes for analysis. Table 1 illustrates the reasons that a physician, insurance company, patient/caregiver, or another pharmacy were contacted. The greatest percentage of notes were call/email/talk to a patient or caregiver, which accounted for 279 of the 322 total notes (~87%). Call/fax to a physician comprised 38 of the total notes evaluated (~12%). The remaining notes (1%) were associated with contacting an insurance company or another pharmacy. The reasons for contacting physicians were further categorized as problems with the prescription itself (15), problems related to cost (7), and medication-related problems (16). The reasons for contacting patients/caregivers were further categorized as education (79), providing information (15), adherence (147), and medication-related problems (38). 

The average number of notes per patient was 1.6. Of the 322 notes, 204 were exclusively medication-related problems. This accounts for 1.02 problems per patient in this two-month study period, which can be extrapolated to 6.12 problem per patient in a 12-month time period. Thirty percent of the total notes reviewed contained actions that were completed by pharmacists. When the 322 notes were further categorized under the MTP categories, 54.7% were related to adherence, 35.4% to safety, 5.6% to effectiveness, and 4.3% to indication. Table 2 describes in detail the notes under each MTP category. 

For the current study, it was determined that the activities categorized under “safety” and “indication” can be linked to a potential adverse drug event and these totaled to 128 activities (Table 2). Keep in mind that the results are limited based on the information available in the notes. For example, the note indicated that the patients were contacted to avoid a potential adverse drug event. However, from the notes, it is not clear whether there was any follow-up activity such as contacting the physician to change the medicine or avert the adverse drug event. Thus, the results manifest potential cost savings associated with possible adverse drug event averts. In other words, even if 10% of these activities resulted in actual avoidance of adverse drug events, it will result in approximately 13 dispensing activities in a two-month span. A careful analysis of these activities from Table 2 will demonstrate that there were nine activities that may have resulted in averting adverse drug event. These include three duplicate therapies, three inappropriate dosages, one allergy/side effect from the prescribed medicine, one medication error from the patient and one medication error from the physician. Burton and colleagues reported the cost associated with each adverse drug event in an ambulatory care setting as $926 in 2006 [9]. Using $1162 as the cost adjusted to 2018 US dollars, these nine activities can result in a health care saving of $10,458 (or a maximum of $148,736 in health care savings if all the 128 activities resulted in averting adverse drug events). Either way, this potential cost saving for 200 patients in a span of two months from one independent pharmacy demonstrates the benefit of these dispensing activities to the patient and the health care system.

## 4. Discussion

The objective of this study was to quantify the additional dispensing activities carried out in a pharmacy to ensure the safe dispensing of a medicine. Though studies have examined the prevalence of medication errors, there was a knowledge gap regarding the additional dispensing activities carried out in a community pharmacy to reduce these medication errors, which is the major strength of this study. While earlier studies had a “patient-focused” approach to determine whether the patient achieved desired therapeutic outcomes after the intervention, this study focused on the “processing” of the prescriptions. The results from this study demonstrated that activities associated with improving adherence and safety were most dominant at the independent pharmacy examined. Most actions that took place involved contact with the patient or caregiver. Dispensing activities related to effectiveness and indication were carried out significantly less when compared to adherence and safety. These results corroborate a study from New Hampshire that examined medication errors in a community pharmacy and concluded that 40% of errors reported involved the incorrect medicine being dispensed; incorrect doses comprised 31% of the errors and 12% were associated with incorrect directions [10]. The Australian Council for Safety and Quality in Health Care also reported selection of the incorrect strength of a medicine or selection of the incorrect product as the cause for most of the errors in the community [11]. A study from England and Wales also reported the most common causes of errors as misreading the prescription, similar drug names, selecting the previous drug or dose from the patient’s medical records, and similar packaging [5]. Recent studies in community pharmacies identified 5.4 medication-related problems per patient and an average of 6.8 interventions per patient in a 12-month study period, which is comparable to the 6.12 problems per patient identified in this study [12,13].

The calculated health care-related costs saved by performing these additional activities from the 200 patients that were evaluated was a significant amount. A previous study (2012) carried out in British Colombia examined cost associated with an “adapted” (dose adjustment, script renewal, therapeutic substitutions, or different formulation) versus a “non-adapted” prescription. On average, an adapted prescription took 6:43 minutes longer to process and had a labor cost of $6.10 more than the non-adapted prescriptions, illustrating the additional time and cost in carrying out the additional dispensing activities [14]. However, the results from this study help demonstrate that the additional time and cost associated with ensuring safe dispensing can result in significant savings. Another study (2013) in Thailand examined the cost savings associated with three additional prescription activities, namely, fixing dosage/frequency, switching intravenous to oral, and eliminating unnecessary medications. The study reported that the total number of pharmacist interventions was 1977 with 253 active recommendations in medication orders, with estimated cost savings of NT$144,138 [15]. This represents cost savings from the whole of 2013 and equates to about $US 4692.93]. In our study, we were able to quantify and examine more than three dispensing activities and it is hard to compare the health care costs between Thailand and the US. 

A substantial literature review was carried out to identify similar studies in the United States. However, the studies identified were limited and had several differing objectives to ours such as exploring the costs of adverse drug events in routine care, evaluating labor costs related to pharmacy adaptation services, and analyzing adverse drug event prevention and cost savings by pharmacist intervention. An evaluation of previous studies helped confirm the deficit of knowledge related to the impact of additional dispensing services in community pharmacies. The results of this study demonstrate the need for quantifying the impact of dispensing services and disseminating it to other stakeholders such as patients, pharmacy management, other health care providers, and payers. At a time when patients demand faster dispensing services and pharmacy management encourage faster turnaround times with dispensing, the results from this study can educate patients and pharmacy management about the efforts that go into each of their prescriptions to ensure that they are safe, effective, and affordable. Similarly, when other health care providers such as physicians recognize the impact of these services, they may be willing to better collaborate with the community pharmacies to provide the best care possible for each patient. Additionally, when payers are taking every step to decrease the health care costs, these studies can demonstrate the need for including community pharmacists and paying them for their efforts. Furthermore, this study demonstrates the need for community pharmacies to have pharmacy management/clinical documentation systems that will make it easier and more efficient for pharmacists to document their patient care activities. If so, one of the limitations of this study could have been avoided had there been better documentation of the "result’ of the pharmacist’s actions. Recent literature describes a new approach in pharmacies to identify medication-related problems, known as “Continuous Medication Monitoring (CoMM)”. This involves systematically assessing each new prescription and refills dispensed for problems, collecting additional information from patients, taking action to resolve identified problems, and importantly documenting problems and actions taken by the pharmacist [12,13].

Limitations of the study: This study has limitations. First, this study was carried out in one independent pharmacy. It is possible that intervention patterns in this specific independent pharmacy differ from other pharmacies and thus are not generalizable. Further studies across other community pharmacies are warranted to be able to more accurately apply the results to all community pharmacies. Next, this study was a retrospective data analysis produced from patient notes. Subsequently, it is unclear whether the annotations resulted in further activities that resulted in increasing patient safety. Additionally, the exact clinical outcome of each individual activity is unknown. For example, a patient may have picked up a refill they were reminded about, but whether they took their medication as prescribed or not was unidentified in this study. Finally, the data collected came from a two-month period. Different times of the year could produce different volumes of prescriptions, thus potentially altering the number of additional dispensing activities performed. Consequently, a longitudinal study with at least one year’s worth of data could be beneficial. Furthermore, as this study was not able to determine the actual results of the pharmacy staff’s actions, the cost savings calculated are potential cost savings, if these assumptions were met. 

## 5. Conclusions

This study was able to show the positive impacts that everyday dispensing services in an independent community pharmacy have on ensuring the safe use of medication, thus potentially preventing adverse drug events and reducing health care costs.

## Figures and Tables

**Table 1:** Reasons for contacting the various agencies for safe prescription dispensing.

Reason for Each Contact	Count (Out of 322 Notes)
**Call/Fax to Physician**	
*Problems with the prescription*	
Incomplete script	3
Refills	6
Medicine recall/backorder	6
*Problems with the cost*	
Lack of insurance coverage	2
Brand to generic	1
Medicine too expensive	2
Formulary coverage	2
*Medication-related problems*	
Inappropriate dosage	3
Inappropriate instructions	0
Drug–drug interactions	0
Allergies/side effects from the prescribed medicine	1
Unnecessary therapy	1
Additional therapy needed	4
Duplicate therapy	3
Medication error	1
New medication	2
Med-list	1
Total for call/fax to physician	38 (11.80%)
**Call/Fax/Email to Insurance Company**	
Prior authorization	1
Early refill	0
Eligibility check	1
Vacation supply	0
Workers compensation	0
Formulary review/process claim	2
Total for call/fax/email to insurance	4 (1.24%)
**Call/Fax to Another Pharmacy**	
Prescription transfer	1
Total for call/fax another pharmacy	1 (0.31%)
**Call/Email/Talk to Patient or Caregiver**	
Education	
Additional education on the medication	65
In-pharmacy counseling	10
Vaccine recommendations	4
Providing information	
Inform of dosage/other change in therapy	10
Communication with home health/courier	2
Communication with patient’s caregiver	3
Adherence	
Med-synch/adherence	80
Reminder to pick up medication (in addition to regular texts)	3
Refill due	64
*Medication-related problems*	
Medication error	1
Prescription clarification	21
Med-list	7
Rx profile review	1
Medication change	8
Total for call/email/talk to patient/caregiver	279 (86.65%)

**Table 2:** Notes under each MTP category.

MTP Category/Reason for Each Contact	Count (Out of 322 Notes)
**Indication**	
Unnecessary therapy	1
Additional therapy needed	4
Duplicate Therapy	3
New medication	2
Vaccine recommendations	4
Total for indication	14 (4.35%)
**Effectiveness**	
Medication change	8
Inform of dosage/other change in therapy	10
Total for effectiveness	18 (5.59%)
**Safety**	
Inappropriate dosage	3
Inappropriate instructions	0
Drug–drug interactions	0
Allergies/side effects from prescribed medicine	1
Physician med-list	1
Additional education on medication	65
Medication error (physician)	1
Medication error (patient)	1
Prescription clarification	21
Patient med-list	7
In-pharmacy counseling	10
Rx profile review	1
Incomplete script	3
Total for Safety	114 (35.40%)
**Adherence**	
Lack of insurance coverage	2
Brand to generic	1
Formulary coverage	2
Medicine too expensive	2
Medicine recall/backorder	6
Prior authorization	1
Early refill	0
Vacation supply	0
Formulary review/process claim	2
Prescription Transfer	1
Med-synch/adherence	80
Reminder to pick up medication (in addition to the regular texts)	3
Refills (physician)	6
Refill due (patient)	64
Communication with home health/currier	2
Communication with patient’s caregiver	3
Eligibility check	1
Workers compensation	0
Total for adherence	176 (54.66%)
